# Short-Term Creatine Loading Improves Total Work and Repetitions to Failure but Not Load–Velocity Characteristics in Strength-Trained Men

**DOI:** 10.3390/nu13030826

**Published:** 2021-03-03

**Authors:** Joshua F. Feuerbacher, Valerian von Schöning, Judith Melcher, Hannah L. Notbohm, Nils Freitag, Moritz Schumann

**Affiliations:** 1 Department of Molecular and Cellular Sports Medicine, German Sport University Cologne, 50933 Cologne, Germany; j.feuerbacher@dshs-koeln.de (J.F.F.); vale_vs@yahoo.de (V.v.S.); judith-melcher@web.de (J.M.); h.notbohm@dshs-koeln.de (H.L.N.); nils.freitag@osp-berlin.de (N.F.); 2 Olympic Training Center Berlin, 13053 Berlin, Germany

**Keywords:** mean propulsive velocity, mean propulsive power, force–velocity profile

## Abstract

This study assessed the effects of a 7-day creatine (CRE) supplementation on the load–velocity profile and repeated sub-maximal bouts in the deep squat using mean propulsive velocity (MPV) and mean propulsive power (MPP). Eleven strength-trained men (31.4 ± 5.4 years) supplemented 0.3 g·kg^−1^·d^−1^ CRE or a placebo (PLA, maltodextrin) for seven days in a randomized order, separated by a 30-day washout period. Prior to and after the supplementation, the subjects performed an incremental maximal strength (1RM) test, as well as 3 × 10 repetitions and a repetitions-to-failure test (RFT), all at 70% 1RM. Maximal strength remained statistically unaltered in CRE (*p* = 0.107) and PLA (*p* = 0.568). No statistical main effect for time (*p* = 0.780) or interaction (*p* = 0.737) was observed for the load–velocity profile. The number of repetitions during RFT remained statistically unaltered in both conditions (CRE: +16.8 ± 32.8%, *p* = 0.112; PLA: +8.2 ± 47.2%, *p* = 0.370), but the effect size was larger in creatine compared to placebo (g = 0.51 vs. g = 0.01). The total work during RFT increased following creatine supplementation (+23.1 ± 35.9%, *p* = 0.043, g = 0.70) but remained statistically unaltered in the placebo condition (+15.0 ± 60.8%, *p* = 0.801, g = 0.08; between conditions: *p* = 0.410, g = 0.25). We showed that CRE loading over seven days did not affect load–velocity characteristics but may have increased total work and power output during submaximal deep squat protocols, as was indicated by moderate effect sizes.

## 1. Introduction

Creatine is a commonly used supplement, especially among strength-trained individuals [1]. Oral creatine intake increases the amount of intramuscular creatine phosphate (PCr) [2], while enhancing the buffer capacity for rapid changes in intramuscular adenosine triphosphate (ATP) and increasing the contribution to ATP resynthesis. Short-term activities of high-intensity are especially dependent on the phosphagen system [3]. PCr and creatine promote the diffusion of high-energy phosphates between their sites of origin (mitochondria) and sites of use (myofibrils, sarcoplasmic membrane). Additionally, PCr hydrolysis counteracts stress-induced acidosis by buffering H^+^ ions [4].

The ergogenic effects of creatine supplementation were previously extensively discussed in multiple systematic reviews and meta-analyses [1,5,6,7,8]. The consensus of these studies is that prolonged creatine supplementation (20 g∙d^−1^) is efficacious in enhancing strength performance (e.g., maximal strength and explosive strength) and anaerobic work capacity. Additional benefits have been observed, such as an increase in body mass and repeated sprint performance [9] as well as muscle fiber hypertrophy and enhanced glycogen levels [8].

In contrast to long-term creatine supplementation, the effects of short-term creatine loading (i.e., 2–7 days) on maximal and explosive strength performance are controversial. Some studies reported increases in maximal [1,10] and explosive strength [1], while others did not find any changes [11]. Moreover, the current literature supports improved anaerobic work capacity (e.g., during a 30 s Wingate test) [9,10,11,12]. Eckerson et al. [12] concluded that a general increase in anaerobic work capacity is linked to maximized creatine stores, which have been reported as early as after two days of loading, therefore highlighting the metabolic benefits of creatine supplementation.

Associations between maximal and explosive strength capacities are best displayed by individual load–velocity profiles [13]. A plethora of studies analyzed load–velocity profiles using a linear velocity transducer in various settings, concluding that velocity-based monitoring of strength performance is a precise method to assess the effort and the estimated relative load of athletes [14,15,16,17]. In addition, the force–power relationship has recently received more attention in helping to maximize power performances in ballistic and multi-joint exercises [18,19,20]. Collectively, these characteristics help to express the maximal neuromuscular abilities in various strength exercises, since the load–velocity–power relationship combines maximal and explosive muscle contractions. However, it remains unknown if and to what extent short-term creatine loading can affect the individual load–velocity and/or the force–power profile. Consequently, the aim of the current study was to assess the effect of a 7-day creatine supplementation on the load–velocity-power relationship using the mean propulsive velocity (MPV). Additionally, velocity-based power measurements were utilized to determine the power outputs and the total work for repetitive submaximal exercise bouts in the deep squat using the mean propulsive power (MPP).

## 2. Materials and Methods

### 2.1. Experimental Design

**Figure 1:**
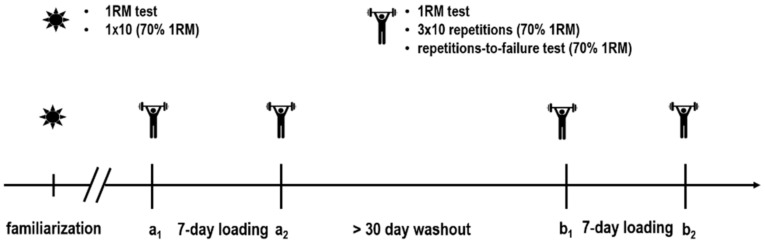
Study design. 1RM = one repetition maximum.

The study design is illustrated in Figure 1. The study comprised a randomized crossover, double-blind, and placebo-controlled trial. Randomization was performed by personnel not involved in data collection.

One week before the initial testing, the subjects underwent a familiarization, consisting of a one repetition maximum test (1RM) and 1 × 10 repetitions (70% of the 1RM), to prevent potential learning effects. After a minimum of 72 h, the subjects returned to the lab for the initial baseline testing (a1). The testing protocol consisted of a 1RM test, 3 × 10 repetitions (70% of the 1RM), and a repetitions-to-failure test (RFT) at 70% of the 1RM. Thereafter, a 7-day intervention period commenced (a1 to a2), during which subjects were given 0.3 g·kg^−1^ per day [21] of either a creatine (CreaZ, B.M.P. Pharma Trading AG, Norderstedt, Germany) or a placebo supplement (Maltodextrin, My Supps GmbH und Co. KG, Ellerbek, Germany), equally divided into four daily servings (creatine: 26.2 ± 2.7 g; placebo: 26.2 ± 2.6 g). Subjects were informed in advance to receive a creatine supplement at each condition. The supplements were prepared in weighed portions by a technician not involved in the data collection. Each serving was consumed with 200 mL apple juice provided by the study personnel. After the 7-day loading period, the a1 testing procedure was repeated (a2). Following the initial intervention period, a washout period of at least 30 days (45 ± 7.1 days) was completed, at the end of which subjects completed the second intervention (b1 to b2). The order of supplementation (i.e., creatine or placebo) was randomized. Before and on the last day of each loading phase, 24 h urine samples were collected. For standardization, subjects were instructed to maintain similar dietary habits and training routines throughout the entire study period. 

### 2.2. Participants

Eleven strength-trained men (age: 31.4 ± 5.4 years, height 180.1 ± 5.8 cm, body mass 87.7 ± 8.8 kg) volunteered to participate in the study. Subjects were recruited through local gyms. The sample size was defined as a priori based on the main outcome, i.e., the load–velocity–power relationship using the mean propulsive velocity (MPV). The projected sample size was assessed by G*Power 3.1. with α = 0.05, an effect size of 0.5 as determined previously [9], and a power of 0.95 [22]. The analysis revealed a required minimum of eight subjects.

Subjects were required to have engaged in regular strength training twice a week for at least three years. Exclusion criteria included any kind of smoking, a vegetarian or vegan diet, shift work, as well as health constraints that would prevent them from performing heavy resistance training. Furthermore, subjects without previous experience in supplementing creatine were excluded. Prior to the start of the study, subjects had to refrain from creatine for at least four weeks. All subjects were informed about the possible risks of the study. Written informed consent was obtained from all subjects before inclusion into the study, and a medical history questionnaire was reviewed. The study was approved by the local ethics committee (011/2020) and was performed in accordance with the Declaration of Helsinki and its later amendments.

### 2.3. Procedures

#### 2.3.1. Strength Testing and Load–Velocity Profiling

After a standardized warm-up, strength testing was conducted to determine the 1RM, as well as the load–velocity profile in the deep squat both at baseline (a1 and b1) and in the morning of day eight after the 7-day loading phase (a2 and b2). Subjects were instructed to attend the testing well-nourished and to keep the food intake similar for each testing protocol. Adherence to these instructions was assured using both a readiness questionnaire and a food diary. Strength testing was performed using a Smith machine (Gym80 international GmbH, Gelsenkirchen, Germany). MPV and MPP values were measured and calculated by utilizing a velocity measurement system (T-Force System, Ergotech, Murcia, Spain), which consists of a cable-extension linear velocity transducer that samples velocity with a rate of 1000 Hz.

The 1RM testing protocol was adapted from González-Badillo and Sánchez-Medina [14]. Strength testing commenced with a warm-up, consisting of five minutes of cycling on a stationary bike at an individually assessed load, followed by unloaded squat jumps, as well as ten unloaded deep squats and one set of six repetitions of deep squats with the unloaded bar (22 kg). Individual foot positioning was determined during the familiarization using tape markings on the floor and was maintained throughout all subsequent experimental testing. The movement pattern for the testing consisted of a deep full squat, followed by a 1.5 s hold at the turning point of the eccentric phase, to avoid utilizing the rebound effect, and an explosive concentric phase to generate maximum velocity. Displacement of the eccentric movement phase was monitored and verified by the tester. The initial load was set at 22 kg (empty bar) and was individually increased after every set of repetitions until the MPV dropped below 0.5 m·s^−1^. Subsequently, the increments were reduced to individually adapted smaller increments (2.5–5 kg) until no further repetition could be performed. A recovery time of two minutes was allowed between the sets. The repetition with the highest load that was executed safely without assistance was regarded as the 1RM. Load–velocity profiles were computed using relative loads (%1RM). The corresponding MPV was calculated using the interpolation method.

#### 2.3.2. Deep Squat Protocol (3 × 10 Repetitions)

After eight minutes of rest, subjects performed a deep squat protocol consisting of three sets with ten repetitions performed at an intensity of 70% of the 1RM. Subjects were instructed to perform the concentric phase as fast as possible. Additionally, they were reminded to hold the deep squat at the turning point of the eccentric phase for 1.5 s to avoid any rebound effects. The rest period between sets was set at two minutes. MPP was recorded consistently throughout the test. The total work was assessed by calculating the area under the curve (AUC) with the integral of the MPP over time using:

AUC = ∫ MPP (t) dt.(1)

#### 2.3.3. Repetitions-to-Failure Test

After an additional eight minutes of rest, subjects performed an RFT. Subjects were instructed to perform as many repetitions as possible with 70% of the 1RM until significant loss of technique became apparent or the subjects were no longer able to perform a deep squat without assistance. Furthermore, during the RFT, subjects were instructed to hold the deep squat at the turning point for 1.5 s and to perform the concentric phase as fast as possible. The total work was assessed by AUC of the MPP.

#### 2.3.4. Urine Sampling

Urine analysis was conducted utilizing the 24 h urine samples before and on the last day of the loading phase. The urine samples were continuously collected for 24 h, beginning with the first voiding in the morning before the testing, and were analyzed for creatinine clearance by a laboratory specialized in routine clinical diagnostics (Labor Dr. Quade, Cologne, Germany).

#### 2.3.5. Statistical Analysis

Data are presented as mean ± SD. All data were analyzed using SPSS 27.0 (SPSS, IBM Statistics, New York, NY, USA). The normality of distribution was assessed by evaluation of q–q plots. The following variables were statistically assessed: body mass and creatinine excretion, 1RM, MPV of each load (i.e., 30–90% 1RM), total work (i.e., AUC of MPP) of each set determined during the 3 × 10 repetitions, total number of repetitions achieved during the RFT as well as total work (i.e., AUC of MPP), and total work normalized per repetition of the RFT. To assess statistical differences between the two conditions (i.e., creatine and placebo), we initially calculated absolute differences within the conditions (i.e., a2-a1 and b2-b1), except for baseline comparisons (i.e., a1 vs. b1). Body mass, creatinine excretion, 1RM, and total numbers of repetitions, as well as total work and total work normalized per repetition in the RFT were compared using a two-tailed *t*-test for dependent samples. The MPV of each load during the 1RM test was assessed by a mixed factorial analysis of variance (ANOVA) using the condition as a between-condition variable and the MPV at every load (i.e., seven loads, 30–90%) as a within-group variable. 

Similarly, total work (i.e., AUC of MPP) in the 3 × 10 repetitions test was assessed by the same method, but using only three time points within groups (i.e., set 1–3). In case of a statistically significant main effect for time or interaction, a Bonferroni post hoc analysis was performed. In case a lack of sphericity was observed in the ANOVA, the Greenhouse–Geisser correction was applied. Statistical significance for all analyses was set at *p* ≤ 0.5. Effect sizes (g) were calculated in order to evaluate the effects of the intervention phases using Hedges g, with g < 0.2 being no effect, g between 0.2 and 0.5 being a small effect, g between 0.5 and 0.8 being a medium effect, and g > 0.8 being a large effect [23,24]. They were calculated for between-condition comparisons, even in the absence of statistical main effects.

## 3. Results

### 3.1. Baseline Comparison

The comparison of baseline values (a1 and b1) for both conditions is displayed in Table 1. No differences were observed in strength and power output or in the load–velocity characteristics.

### 3.2. Maximal Strength and Load–Velocity Characteristics

**Figure 2:**
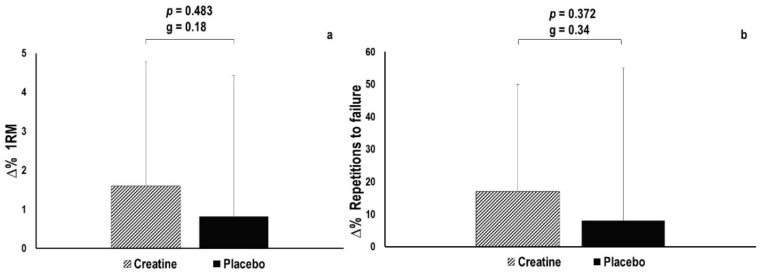
(**a**) Changes in maximal strength (one repetition maximum, 1RM) after a 7-day ingestion of creatine or placebo supplement. (**b**) Changes in number of repetitions performed in the repetitions-to-failure test (RFT) test after a 7-day loading of creatine or placebo.

The maximal strength remained statistically unaltered in both the creatine (+1.6 ± 3.2%, i.e., increase in strength from 134.5 ± 20.9 kg to 136.8 ± 22.1 kg, *p* = 0.107) and placebo condition (0.8 ± 3.6%, i.e., increase in strength from 135.2 ± 21.6 kg to 136.1 ± 21.1 kg, *p* = 0.568) (Figure 2a). No statistically significant between-condition differences were observed (*p* = 0.483, g = 0.18). However, the effect size in the creatine condition was larger than that observed in the placebo condition (g = 0.52 vs. g = 0.21). Relative maximal strength remained statistically unaltered in both the creatine (+0.4 ± 4.2%, i.e., increase in relative strength from 1.5 ± 0.2 kg·kg^−1^ to 1.6 ± 0.2 kg·kg^−1^, *p* = 0.724, g = 0.11) and placebo condition (0.4 ± 5.1%, i.e., increase in relative strength from 1.5 ± 0.2 kg·kg^−1^ to 1.6 ± 0.2 kg·kg^−1^, *p* = 0.192, g = 0.41). No statistically significant between-condition differences were observed (*p* = 0.727, g = 0.10).

**Figure 3:**
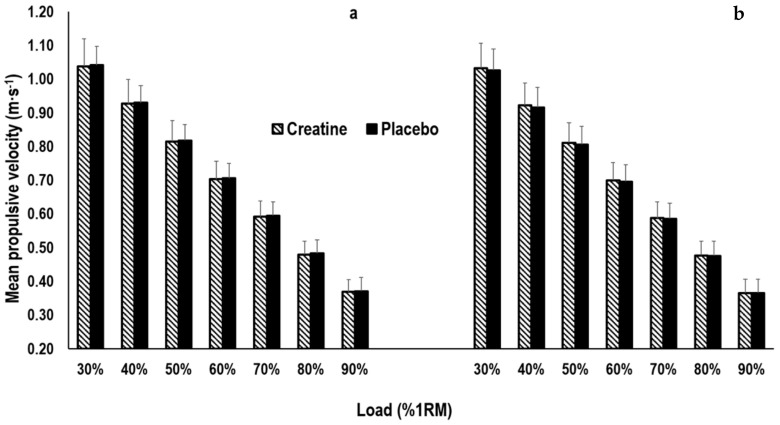
Changes in the load–velocity profile for the creatine and placebo condition pre (**a**) and post (**b**) of the 7–day supplementation period. %1RM = relative load of one repetition maximum.

No statistically significant main effects were observed for time (F(1.000, 20.004) = 0.307, *p* = 0.585) at each relative load or interaction (F(1.000, 20.004) = 0.113, *p* = 0.740) between the creatine and placebo condition in the load–velocity profile (Figure 3), while the effect sizes for interaction between the conditions were small and ranged from g = 0.07 to g = 0.13.

### 3.3. Deep Squat Protocol (3 × 10 Repetitions)

**Figure 4:**
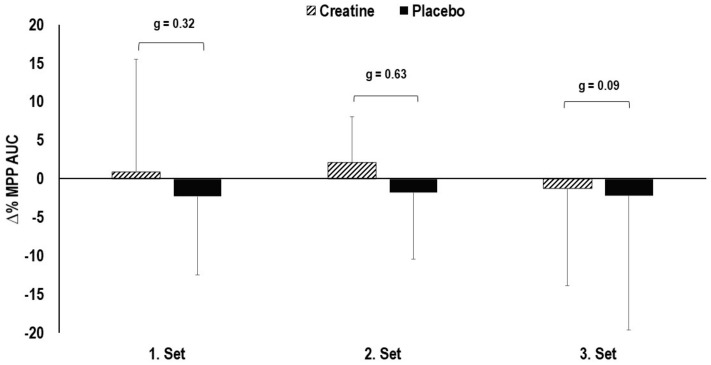
Changes in total work in the 3 × 10 deep squat protocol. MPP = mean propulsive power; AUC = area under the curve.

No statistically significant main effects in total work in the 3 × 10 deep squat protocol were observed for time (i.e., between the three sets) (F (1.931, 38.614) = 0.428, *p* = 0.648) or interaction (F(1.931, 38.614) = 0.236, *p* = 0.783) between the creatine and placebo condition (Figure 4). The effect sizes indicated a small and moderate between-condition effect for the first and second set (g = 0.32 and g = 0.63, respectively).

### 3.4. Repetitions-to-Failure Test

The total number of repetitions performed in the RFT remained statistically unchanged, in both the creatine (+16.8 ± 32.8%, i.e., increase from 10.9 ± 3.4 to 12.6 ± 5.1 repetitions, *p* = 0.112) and placebo condition (+8.2 ± 47.2%, i.e., increase from 11.2 ± 4.5 to 11.3 ± 5.3 repetitions, *p* = 0.949) (Figure 2b). No statistically significant between-condition differences were observed (*p* = 0.372, g = 0.34); however, the effect size was larger in creatine compared to the placebo condition (g = 0.51 vs. g = 0.01).

**Figure 5:**
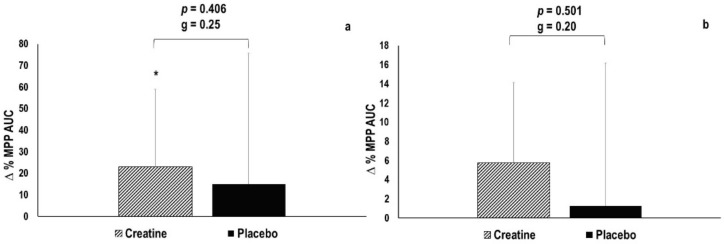
(**a**) Changes in total work during repetitions-to-failure test (RFT). (**b**) Changes in total work per repetitions performed in RFT. MPP = mean propulsive power; AUC = area under the curve; * Statistically significant difference between pre and post, *p* ≤ 0.05.

The total work achieved during the RFT improved statistically significant by +23.1 ± 35.9% in the creatine condition (+786.3 ± 1062.5 J, *p* = 0.043, g = 0.70), while it remained statistically unaltered in the placebo condition (+15.0 ± 60.8%, +149.7 ± 1825.5 J, *p* = 0.801, g = 0.08) (Figure 5a). No statistically significant between-condition difference was observed (*p* = 0.406, g = 0.25). MPP normalized per repetition during the RFT remained statistically unaltered in both the creatine (+5.1 ± 9.1%, +15.5 ± 27.9 J per repetition, *p* = 0.095) and placebo condition (+1.0 ± 14.9%, +2.9 ± 47.9 J per repetition, *p* = 0.852) (Figure 5b). No statistically significant between-condition difference was observed (*p* = 0.501, g = 0.20). However, the effect size in the creatine condition was larger than that observed in the placebo condition (g = 0.54 vs. g = 0.02).

### 3.5. Body Mass and Creatinine Excretion

The body mass increase was statistically significant in the creatine condition by +1.2 ± 1.4% (+1.0 ± 1.2 kg, *p* = 0.016, g = 0.83) and decreased statistically in the placebo condition (−0.5 ± 0.8%, −0.4 ± 0.7 kg, *p* = 0.006, g = 1.0). No statistically significant between-condition differences were found for body mass, but the effect size indicated a moderate effect (*p* = 0.058, g = 0.57). The urine analysis showed no statistical change in creatinine excretion in the creatine (+36.4 ± 92.7%, +0.18 ± 0.62 g·L^−1^, *p* = 0.32, g = 0.31) and placebo conditions (+3.9 ± 35.7%, +0.14 ± 0.44 g·L^−1^, *p* = 0.323, g = 0.29), but the between-condition effect size indicated a large effect (*p* = 0.236, g = 0.88).

## 4. Discussion

The present study evaluated the effects of a 7-day creatine supplementation (0.3 g·kg^−1^·d^−1^) on the load–velocity profile using the MPV. Additionally, power output in the repeated deep squat protocol and RFT test was assessed. Our findings indicate that short-term creatine supplementation does not affect the load–velocity profile of the incremental 1RM test. However, creatine supplementation led to statistically improved mean total work during the RFT, while it was maintained in the placebo condition. Similarly, the larger effect sizes for mean power normalized per repetition and the total number of repetitions in the RFT in the creatine condition indicate further beneficial effects. Additionally, creatine supplementation led to improvements in total work in the repeated deep squat protocol (3 × 10 repetitions at 70% of 1RM), as was indicated by the small and moderate effect sizes in the first two sets.

Creatine supplementation did not enhance the neuromuscular ability to generate more force at a given relative load. Hence, the main finding of the present study was that creatine did not induce increases in the load–velocity relationship. While marginal gains in maximal strength were reported (+1.6 ± 3.2%), the MPV did not change at any load of the load–velocity profile. This underlines the fact that creatine supplementation does not affect single bouts of maximal muscle contraction, since these efforts do not predominantly depend on the phosphagen system [25]. Marginal increases in maximal strength may have two possible explanations. Firstly, due to the 1RM tests being performed before and immediately after the 7-day supplementation period, it was impossible to attain blind results in maximal strength. Therefore, the motivational factors of the subjects wanting to outperform themselves may have had an impact. Secondly, creatine is known to improve the resynthesis of ATP, and since the subjects executed at least eight sets in the incremental 1RM test, this may possibly have enhanced their performance towards the end.

Several studies have reported increases in maximal strength after 5–7 days of creatine supplementation (20 g∙d^−1^) [9,10,26]. However, according to Izquierdo and colleagues [9], these improvements also depend on the type of the maximal strength test performed. Longer contraction times imply a greater impact on the phosphagen system [3]. This might explain the increases in maximal strength in studies that have assessed maximal strength through isometric contractions, lasting up to 3–5 s [26], or repetitive (3–4 repetitions per load) 1RM trials [10]. In contrast, the present study utilized a single-repetition protocol, where each repetition was performed with the highest velocity possible. Additionally, individual characteristics need to be considered. Some subjects respond to creatine supplementation differently due to diminished PCr storage and no previous creatine supplementation experience. Hence, adaptations in strength performance differ between subjects who have previously engaged in creatine supplementation and subjects who have not, due to lower adaptation capacities [27]. To minimize these effects, the present study only included trained subjects that had previously engaged in creatine supplementation. However, subjects were instructed to refrain from creatine intake for at least 30 days prior to the two loading phases.

In order to investigate whether creatine loading enhances explosive strength in single exercise bouts, the present study included velocity-based measurements throughout the repeated submaximal deep squat intervention and the RFT. Interestingly, statistical improvements were reported for total work in the RFT following creatine supplementation only (+23.1 ± 35.9%, *p* = 0.043), mainly due to the larger number of repetitions performed. However, no changes in power output were reported in the 3 × 10 protocol, but mean power per repetition increased by +5.1 ± 9.1%, even though the difference was not statistically significant (*p* = 0.095, g = 0.5). This finding is well in line with previous studies reporting increased power outputs in repetitive bouts of exercise [9,26,28,29,30]. Thus, by assessing the MPP, the findings of our study expand on previous findings that short-term creatine supplementation enhances force production and power output in strength exercises, due to delayed onset of fatigue but does not alter explosive strength in single deep squat bouts.

Our findings are also supported by previous reports, which showed that creatine supplementation leads to increased power output due to improved ATP regeneration, since it enlarges the PCr pool within the muscle fibers [25]. Hence, PCr buffers rapid changes in intramuscular ATP concentration after short-term, high-intensity exercise, while PCr hydrolysis also counteracts stress-induced acidosis by buffering H^+^ ions [4], both of which enhance fatigue resistance. The 3 × 10 protocol, and especially the RFT, were performed in a fatigued state, since they were performed after the incremental 1RM test and the 3 × 10 deep squat protocol, respectively. Likely as a result of this increasing fatigue, the greatest improvements in MPP and total work were reported in the RFT, where typically the greatest influence of previous loading on the deep squat performance exists. These findings underline the beneficial effects of creatine on fatiguing strength protocols and are supported by findings of Izquierdo and colleagues [9], who showed that creatine supplementation reduces the fatigue-related decline in explosive strength.

The beneficial effects of creatine supplementation were also emphasized by statistical increases in body mass and the moderate between-condition effect size for creatinine excretion, indicating that creatine supplementation likely led to increased intracellular PCr content. Indeed, short-term gains in body mass are typically related to water retention within the muscle [31]. Hence, both the increase in body mass and creatinine clearance indirectly indicate that creatine supplementation was successful in improving creatine storage in the muscle fibers, as has been well documented after creatine supplementation [9,10,12,32]. It could be speculated that increases in body mass may also lead to enhanced power output in the deep squat protocols. However, since maximal strength and MPP in the 3 × 10 protocol remained statistically unchanged following creatine supplementation, it can be assumed that increases in body mass were unlikely to have contributed to the observed improvements in the RFT. Furthermore, it is important to bear in mind that creatinine excretion is an indirect marker of the intracellular creatine levels and may not directly reflect creatine metabolism [33].

## 5. Conclusions

We showed that creatine loading with 0.3 g·kg^−1^·d^−1^ for seven days had no impact on the load–velocity characteristics (e.g., maximal and explosive strength) in strength-trained individuals. However, based on moderate between-condition effect sizes, we conclude that creatine supplementation may improve MPP during a repeated deep squat protocol (3 × 10 repetitions) after the 7-day loading. Furthermore, creatine supplementation may improve mean total work, total work normalized per repetition, and total number of repetitions during a RFT that is performed in a fatigued condition. Future research should assess whether long-term creatine supplementation accompanied by strength training is efficient in enhancing maximal and explosive strength and can therefore improve load–velocity characteristics of athletes.

## Figures and Tables

**Table 1:** Pre-test values for the randomized conditions. Values are presented as mean ± SD. 1RM = one repetition maximum; MPV = mean propulsive velocity; RFT = repetitions-to-failure test.

	Pre-Creatine	Pre-Placebo	*p*-Value, g
Absolute 1RM (kg)	135.2 ± 21.6	134.5 ± 20.9	0.524, 0.20
Relative 1RM (kg·kg^−1^)	1.5 ± 0.2	1.5 ± 0.2	0.588, 0.16
Load–velocity characteristics (regression equation of load and MPV in m·s^−1^)	f(MPV) = (–0.008x) + 1.37	f(MPV) = (–0.009x) + 1.38	0.995, <0.08
Total work (3 × 10 repetitions) (J)	3363.5 ± 649.1	3403.9 ± 799.8	0.524, 0.19
Repetitions to failure (RFT)	10.9 ± 3.6	11.2 ± 4.0	0.732, 0.10
Total work (RFT) (J)	3435.6 ± 1340.2	3656.7 ± 1849.8	0.371, 0.27
MPP normalized per repetition (RFT) (J)	313.9 ± 58.7	321.3 ± 76.7	0.394, 0.26

## Data Availability

The data presented in this study are available on request from the corresponding author. The data are not publicly available due to privacy and ethical restrictions.
